# Different neural substrates for precision stepping and fast online step adjustments in youth

**DOI:** 10.1007/s00429-017-1586-9

**Published:** 2018-01-24

**Authors:** Sharissa H. A. Corporaal, Sjoerd M. Bruijn, Wouter Hoogkamer, Sima Chalavi, Matthieu P. Boisgontier, Jacques Duysens, Stephan P. Swinnen, Jolien Gooijers

**Affiliations:** 1 Movement Control and Neuroplasticity Research Group, Department of Movement Sciences, KU Leuven, Tervuursevest 101, box 1501, 3001 Leuven, Belgium; 2 Department of Human Movement Sciences, MOVE Research Institute Amsterdam, VU University Amsterdam, Amsterdam, The Netherlands; 3 Department of Integrative Physiology, University of Colorado, Boulder, USA; 4 Leuven Research Institute for Neuroscience and Disease (LIND), KU Leuven, Leuven, Belgium

**Keywords:** Childhood development, Adolescence, Brain, White matter, Precision stepping, Locomotion

## Abstract

Humans can navigate through challenging environments (e.g., cluttered or uneven terrains) by modifying their preferred gait pattern (e.g., step length, step width, or speed). Growing behavioral and neuroimaging evidence suggests that the ability to modify preferred step patterns requires the recruitment of cognitive resources. In children, it is argued that prolonged development of complex gait is related to the ongoing development of involved brain regions, but this has not been directly investigated yet. Here, we aimed to elucidate the relationship between structural brain properties and complex gait in youth aged 9–18 years. We used volumetric analyses of cortical grey matter (GM) and whole-brain voxelwise statistical analyses of white matter (WM), and utilized a treadmill-based precision stepping task to investigate complex gait. Moreover, precision stepping was performed on step targets which were either unperturbed or perturbed (i.e., unexpectedly shifting to a new location). Our main findings revealed that larger unperturbed precision step error was associated with decreased WM microstructural organization of tracts that are particularly associated with attentional and visual processing functions. These results strengthen the hypothesis that precision stepping on unperturbed step targets is driven by cortical processes. In contrast, no significant correlations were found between perturbed precision stepping and cortical structures, indicating that other (neural) mechanisms may be more important for this type of stepping.

## Introduction

During childhood, walking (gait) skills are developed, going from independent but very unstable gait at infancy (10–18 months of age; Forssberg 1985), to adult-like gait patterns around 8 years of age. This is reflected by matured step speed, step length, stride length, and temporal variability of step cycle duration (Hausdorff et al. 1999; Vaughan et al. 2003; Dusing and Thorpe 2007; Froehle et al. 2013). This holds, however, only for simple forms of gait (i.e., ‘basic gait’), in which the preferred gait pattern can be maintained. Basic gait is rare in everyday life, as humans normally walk through challenging environments (e.g., cluttered or uneven terrains), possibly with obstacles that force the modification of the preferred gait pattern (i.e., ‘complex gait’). Studies illustrate an ongoing refinement of complex gait strategies after the age of 8 years during complex gait tasks such as obstacle avoidance, precision stepping, and dual-task walking. This refinement is reflected by improving gait speed and obstacle clearance (Pryde et al. 1997; Michel et al. 2010), the presence of adult-like muscle activations (McFadyen et al. 2001), and more efficient foot placements (Berard and Vallis 2006; Choi et al. 2016; Corporaal et al. 2016). It has been suggested, but not established, that this extended refinement relates to ongoing neural maturation of cognitive processes underlying the control of complex gait (e.g., Pryde et al. 1997; Choi et al. 2016; Corporaal et al. 2016).

A recent systematic review on brain activation during imagined walking using MRI/fMRI or during real walking using measurement systems such as fNIRS, EEG, and PET (Hamacher et al. 2015) gave an extensive overview of brain areas involved during walking. Although a large network of brain areas is active during walking and walking imagery compared to standing [e.g., supplementary motor area (SMA), primary motor cortex, prefrontal cortex, premotor cortex, cingulate cortex, temporal gyrus, occipital cortex, parietal area, (pre)cuneus, thalamus, parahippocampal gyrus, putamen, globus pallidus, mesencephalic locomotor region, and cerebellum], the main message of this review article is that the level of involvement of the various brain regions depends on the specific task, its level of complexity, patient’s pathology and/or participant's age. Specifically, gait tasks with a higher complexity level are more likely to be associated with increased activation in prefrontal areas, SMA and areas involved in multisensory processing (Hamacher et al. 2015). Thus, a wide range of cortical areas should probably be matured/developed to perform skilled, complex gait.

Considering the structural maturation of GM brain regions which are involved during gait and their WM connections, it has been shown that higher order brain regions (e.g., prefrontal cortices associated with complex gait) generally mature later than lower order brain regions (e.g., sensorimotor cortices associated with basic gait) (Sowell et al. 1999; Gogtay et al. 2004; Casey et al. 2005; Paus 2005; Kochunov et al. 2012; Ducharme et al. 2016). Although the exact age at which both maturation processes (WM and GM) are completed remains unclear, they appear to evolve until around 30 years of age (Giedd et al. 1999; Lenroot and Giedd 2006; Ostby et al. 2009; Lebel and Beaulieu 2011; Raznahan et al. 2011, 2014; Yap et al. 2013; Herting et al. 2015; Ducharme et al. 2016). In general, such developmental brain changes are characterized by decreasing GM volume and increasing WM volume, which is likely related to biological processes such as synaptic pruning and myelination, respectively (Huttenlocher 1990; Giedd et al. 1999; Lenroot and Giedd 2006; Petanjek et al. 2008; Yap et al. 2013; Kostovic et al. 2014).

These hierarchical maturation patterns of higher versus lower level brain regions seem to concur with the development of complex versus basic gait. Although studies in children are lacking, studies in older adults have illustrated the impact of age-related structural brain decline on gait. For instance, smaller GM volumes of sensorimotor and frontoparietal regions were associated with shorter steps and longer double support times (Rosano et al. 2008), smaller prefrontal GM volumes with lower gait speed (Rosano et al. 2012), and smaller parietal GM volume with larger variability of stride length (Beauchet et al. 2014). In addition, disrupted WM properties have been associated with poorer gait performance in older adults (Bhadelia et al. 2009; de Laat et al. 2011; Koo et al. 2012; Bruijn et al. 2014). For example, decreased microstructural organization of the corticospinal tract and thalamic radiation was related to decreased gait stability (Bruijn et al. 2014), and decreased organization of the genu of the corpus callosum with decreased gait performance, as measured with Tinetti gait scores (Bhadelia et al. 2009). Translating these findings to childhood development, it may be possible that immature brain structures impact complex gait performance in children.

Therefore, the primary aim of the present study was to investigate the impact of structural brain changes on complex gait skills in youth aged 9–18 years. We challenged gait control by projecting step targets onto a treadmill, similar to tests used by other members of our group (Potocanac et al. 2014; Hoogkamer et al. 2015; Mazaheri et al. 2015). The complexity of precision stepping was manipulated by occasionally and unexpectedly shifting step targets to new locations during the execution phase of the step. On a behavioral level, we hypothesized that step accuracy would improve with age, yet deteriorate with higher levels of complexity. This would be reflected by decreasing step error and step variability with age, and increasing step error and step variability for the higher levels of complexity. Combining brain and behavior, we hypothesized that step accuracy would improve with whole-brain structural maturation (decreased GM volume, increased WM microstructural organization). We also explored whether the maturation of higher order brain areas involved in motor planning and attention (e.g., frontal areas) are relevant for precision stepping performances. Moreover, these neurobehavioral relationships were assumed to be more pronounced with increasing task complexity. This study is a preliminary step towards understanding the structural neural underpinnings of complex sensorimotor tasks.

## Methods

### Participants

Thirty participants were included for behavioral data analyses (13 females; age range 9.0–18.5 years; mean age 14.4 ± 2.6 years). All participants received a brain scan for GM and WM analyses. For both GM (*N* = 28, 13 females, age range 9.0–17.9; mean age 14.2 ± 2.6), and WM analyses (*N* = 28, 13 females, age range 9.0–18.4; mean age 14.5 ± 2.6), two (different) male participants were excluded due to excessive head motion and/or technical problems during imaging acquisition. All participants were right-handed as indicated by the Oldfield Handedness scale (Oldfield 1971) (mean: 0.79, SD: 0.19, range: 0.08-1). Most participants reported a right-foot preference, although one participant (15.83 years, male) reported a left-foot preference, and three participants (14.43 years, female; 15.77 years, female; 11.64 years, male) reported ambiguous foot preference. Participants reported no neurological, muscular, or cognitive disorders, and were screened for MRI compatibility (i.e., no MRI-incompatible implants, dental braces, and claustrophobia). All procedures performed were in accordance with the ethical standards of the local ethics committee of the KU Leuven, Belgium, and with the 1964 Helsinki declaration and its later amendments or comparable ethical standards. Informed consent was obtained from all individual participants included in the study and their parents. Participants were financially compensated for participation.

### Experimental setup

The participants walked on an instrumented treadmill with an embedded force platform (C-Mill, ForceLink, Culemburg, The Netherlands) allowing for online detection of gait events based on ground reaction forces (1000 Hz sampling rate) (Roerdink et al. 2008). The treadmill was equipped with a projector (Hitachi CP-A100) to present step targets onto the walking surface, adjusted to the participants’ preferred gait pattern (van Ooijen et al. 2013; Potocanac et al. 2014; Hoogkamer et al. 2015; Mazaheri et al. 2015). Furthermore, ten infrared cameras (Vicon Nexus version 1.8.5; 150 samples/s) registered the position of six reflective markers, which were attached to the shoes of the participant, on locations corresponding to the third metatarsal head, lateral malleolus, and heel of each foot. During the experiment, participants wore a safety harness that was fixed to the ceiling to protect them from falling.

### Procedure

**Fig. 1:**
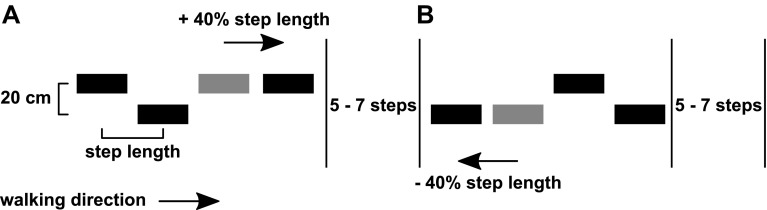
Schematic overview of the step targets and their shifts in forward–backward direction (Adapted from Mazaheri et al. 2015). The distance between the step targets was determined based on participant’s preferred step length. At random moments (separated by 5–7 non-shifted steps), a step target could shift forwards (+ 40% of step length) or backwards (− 40% of step length), requiring a longer step (**a**) or shorter-step (**b**) response. The shift targeted either the right or left leg

After a familiarization period of 5–10 min at 3 km/h of treadmill walking, in which no step targets were projected, ground reaction forces were obtained from 20 normal strides at 3 km/h. From these forces, the center of pressure (COP) trajectory was calculated, and used to extract foot-strike events, toe-off events, and step lengths (Roerdink et al. 2008). The obtained step length represented the ‘preferred’ step length during unperturbed treadmill walking, which was subsequently used to set the anteroposterior center-to-center distance between step targets in the experimental trials. During these experimental trials, participants were required to step as accurately as possible on the step targets projected on the treadmill. The step targets were scaled to the size of the shoe (shoe length by shoe width). Due to technical constraints (use of split belt), the mediolateral center-to-center distance between the targets was set to 20 cm for all participants (Fig. 1). None of the participants reported problems with these step widths.

Projection of the step targets started at 2 m in front of the participants, and approached the participants corresponding to the belt speed (3 km/h). Participants were instructed to place their feet on the step targets as accurately as possible. Participants performed a maximum of two practice trials to familiarize themselves with the precision stepping procedure (59 step targets per trial).

During the experiment, four blocks of step targets were randomly presented. One block consisted of a series of 59 non-shifting step targets, allowing the assessment of stepping precision on unperturbed step targets. In the remaining three blocks, a series of 248 step targets each was presented. Some of these targets occasionally shifted to a predefined new position corresponding to 40% of the preferred step length forwards or backwards (perturbed precision stepping) (Fig. 1). Shifted targets were presented for both the left and right foot and were separated by five-to-seven non-shifted targets. Forward shifts could occur when the target was at different distances from the participant’s COP, resulting in four “available response distances” (ARD) conditions of 80, 100, 130, and 200% of the preferred step length (Potocanac et al. 2014; Hoogkamer et al. 2015; Mazaheri et al. 2015). The shorter the ARDs were, the faster the step corrections had to be made. We, therefore, assumed that shorter ARDs increased the difficulty of making accurate step adjustments (Hoogkamer et al. 2015). Backward shifts only occurred at ARD of 130%, and were solely used to limit anticipation to the direction of the gait perturbations. Hence, backward shifts were not analyzed. Each ARD shift was repeated 8 times (4 per foot), with a total of 5 (4 forwards + 1 backwards) × 8 = 40 target shifts per block. Participants were told in advance whether the block contained shifting targets or not, but were naïve to the exact timing of these perturbations.

### Data analyses

Stepping accuracy on step targets was defined by two error measures, namely, the constant error (*step error*) and variability (*step variability*). *Step error* was defined as the anteroposterior distance between the centers of the targeting foot and the step target at midstance. First, the moment of midstance was obtained during the single stance phase of the foot, i.e., at 50% between toe off and heel strike of the contralateral foot. Second, the center of the foot was defined using the 3D coordinates of the foot markers at the moment of midstance. The lines connecting the foot markers at the heel, toe, and malleolus were used as a representation of the foot at midstance. Subsequently, the center of these connecting lines was defined as the center of the foot. The location of the step target’s center at the moment of midstance was extracted from the C-mill software. Positive error measures indicated overshoots of the step targets (i.e., the center of the foot landed anterior to the center of the step target), while negative values indicated undershoots of the step targets (i.e., the center of the foot landed posterior to the center of the step target).

For unperturbed precision stepping, the step errors of all unperturbed steps within the unperturbed trials were averaged and subsequently normalized to preferred step length, resulting in the *unperturbed step error*. For perturbed precision stepping, the step errors of perturbed steps within the perturbed trials were averaged (per ARD condition) and subsequently normalized to preferred step length, resulting in the *perturbed step error*.

In addition, we calculated the *variability* of both unperturbed and perturbed steps according to the following formula:

$${\text{Variability}}=~\sqrt {\frac{{\Sigma ~{\text{step}}\;{\text{erro}}{{\text{r}}^2} - \frac{{{{\left( {\Sigma ~{\text{step}}\;{\text{error}}} \right)}^2}}}{k}}}{k}} ~\% ~{\text{preferred}}\;{\text{step}}\;{\text{length}}$$

in which *step error* represents the step error per step (non-averaged and non-corrected for step length) in each condition, and k is the number of steps analyzed. Variability was expressed as the percentage of preferred step length.

### Image acquisition and analyses

#### Image acquisition

A Philips Ingenia 3T CX MRI scanner with a standard 32 channel head coil was used for image acquisition. For all participants, a high-resolution T1-weighted structural image was acquired using MPRAGE (TR = 9.71 ms, TE = 4.60 ms, 0.98 × 0.98 × 1 mm^3^ voxels, field of view = 210.94 × 230 mm^2^, 230 sagittal slices) for anatomical detail. In addition, single-shell diffusion-weighted images were acquired using the following parameters: single-shot spin echo; slice thickness = 2.5 mm; TR = 7600 ms; TE = 65 ms, number of diffusion directions = 60, number of sagittal slices = 58, voxel size = 2.5 × 2.5 × 2.5 mm^3^; diffusion weighting of *b* = 1300; one non-diffusion-weighted image.

#### Cortical grey matter: volume processing

Cortical reconstruction and volumetric segmentation were performed using the FreeSurfer image analysis suite (v5.1; http://surfer.nmr.mgh.harvard.edu/). From the T1-weighted images, cortical volume (mm^3^) measures were extracted. Details of these procedures were described in prior publications (Dale et al. 1999; Fischl and Dale 2000; Fischl et al. 2002, 2004a, b). Briefly, this procedure included motion correction of the raw T1-weighted images (Reuter et al. 2010), brain extraction, and Talairach transformation. Then, WM and GM were segmented (Fischl and Dale 2000; Fischl et al. 2004a) and intensity inhomogeneities were normalized (Sled et al. 1998). The GM/WM boundary was tessellated and the surface was deformed following intensity gradients to optimally place the GM/WM and GM/cerebrospinal fluids borders at the location, where the greatest shift in intensity defines the transition to the other tissue class (Dale and Sereno 1993; Dale et al. 1999; Fischl and Dale 2000). Once the cortical models were completed, a refinement procedure was applied to obtain a representation of the GM/WM boundary. This surface was subsequently deformed outwards to obtain an explicit representation of the pial surface, which was then divided into distinct cortical regions. The parcellation procedure labeled cortical sulci and gyri to 68 cortical regions (i.e., ‘parcels’) (Fischl et al. 2004b; Desikan et al. 2006), from which cortical volumes were calculated. Cortical volumes of the frontal, temporal, parietal, occipital, and insular cortex (i.e., ‘superparcels’) of both the left and right hemisphere were calculated as the sum of the volumes of each parcel falling within each superparcel (Table 1).

**Table 1:** Superparcel formation for cortical GM measurements (used with approval of Chalavi et al. 2015)

Superparcel name	Cortical parcels
Frontal	Caudal and rostral middle frontal, lateral and medial orbito frontal, pars opercularis, pars triangularis and pars orbitalis of the inferior frontal, paracentral, precentral, superior frontal, frontal pole, caudal and rostral anterior cingulate
Parietal	Superior and inferior parietal, postcentral, precuneus, isthmus and posterior cingulate, supramarginal
Temporal	Superior, middle and inferior temporal, temporal pole, transverse temporal, banks of the superior temporal sulcus, parahippocampal, entorhinal, fusiform
Occipital	Lateral occipital, cuneus, lingual, peri-calcarine
Insula	Insula
Whole-brain	Sum of the five superparcels (Frontal, Parietal, Temporal, Occipital, Insula)

#### White matter: diffusion—weighted image processing

We performed quality checks on each diffusion-weighted imaging volume using Explore DTI (Leemans 2009). As recommended by Tournier et al. (2011), we inspected the volumes in three orthogonal views (sagittal, coronal, and frontal) to identify visible artifacts, such as large signal dropouts and geometric distortions. When an artifact was detected in an isolated volume, this volume was removed. For seven participants, one to a maximum of five isolated volumes was removed. One participant moved the head with more than 2 mm translation and/or 2° rotation in the 17th assembled volume. Therefore, all prior assembled volumes were removed (16/61) before the movement onset, making sure that at least 2/3 of all volumes remained.

After the quality checks, diffusion-weighted data were further preprocessed using the FMRIB (Functional MRI of the Brain) Software Library, FSL (Oxford University, Oxford, UK; http://www.fmrib.ox.ac.uk/fsl) (Smith et al. 2004; Woolrich et al. 2009; Jenkinson et al. 2012). For each participant, eddy-current-induced geometric distortions and head movements were corrected. Then, the diffusion-weighted volumes were corrected for distortions as a result of magnetic field inhomogeneities using fieldmap correction and were aligned to their corresponding non-diffusion-weighted (b0) image. The gradient direction table was adjusted to account for rigid transformations resulting from motion and eddy-current corrections. Subsequently, the diffusion-weighted images were brain-extracted using BET (Smith 2002), and a diffusion tensor model was fitted to each voxel using DTIfit procedure of the FMRIB’s Diffusion Toolbox. This procedure outputs whole-brain FA and MD images, and additionally provides three eigenvalues of the diffusion tensor model (ʎ1, ʎ2, and ʎ3). From these eigenvalues, images of apparent diffusivities in the directions parallel (i.e., axial diffusivity (AD) = ʎ1) and perpendicular (i.e., radial diffusivity (RD) = (ʎ2 + ʎ3)/2) to the WM tracts were created (Kumar et al. 2013).

### Statistical analysis

#### Behavioral analyses

For unperturbed precision stepping trials, age-related changes in *unperturbed step error* and *variability* were assessed via bivariate Pearson correlation analyses (two-tailed). For perturbed trials, we additionally included the effect of ARD on perturbed step error and variability; hence, a repeated measures ANCOVA with age as a covariate and ARD as a within-subject factor was used. Level of significance was set at *p* < 0.05 for all statistical tests.

#### Imaging analyses

##### Grey matter

Associations between volumes of cortical GM regions of interest (ROIs) from both hemispheres (i.e., the frontal, parietal, temporal, occipital and insular superparcels, as well as all the 68 parcels), and *step error* and *variability* of unperturbed and perturbed steps, were assessed using Pearson correlations. Since it has been proposed that approximately 95% of maximal brain size is reached by the age 6 years, we assumed that differences in GM volume were most likely due to ongoing cortical maturational processes, rather than normal physical growth (Lenroot and Giedd 2006). However, to account for such a possible underlying process, we additionally performed the correlations while correcting for total intracranial volume (ICV). Furthermore, we tested the correlations while controlling for age, to identify possible variance in the relationship that could not be accounted for by age. The resulting *p* values were corrected for multiple comparisons using the false discovery rate (FDR; *q* < 0.05) per dependent variable (*unperturbed* and *perturbed* step error and *variability*) and including all superparcels (frontal, parietal, temporal, occipital, and insula) or the parcels belonging to each superparcel, per hemisphere (Benjamini and Cohen 2017; Drijkoningen et al. 2017).

##### White matter

Voxelwise statistical analyses of the FA, MD, RD, and AD measures were performed using TBSS (Tract-Based Spatial Statistics; Smith et al. 2006), part of FSL (Smith et al. 2004). Our rationale for applying TBSS was its robustness to systematic differences between participants, such as differences in brain size and developmental stage. It allows for statistical testing which is less affected by potential misalignment, and thereby provides more objective results (Smith et al. 2006). This method is appropriate for the analysis of large white-matter bundles, thereby excluding superficial white matter consisting of short-range association bundles. All participants’ FA data were aligned to a common space using the nonlinear registration tool FNIRT (Smith et al. 2004; Woolrich et al. 2009; Jenkinson et al. 2012), which uses a b-spline representation of the registration warp field (Rueckert et al. 1999). The mean FA image was created and thinned to create a mean FA skeleton which represents the centers of all tracts common to the group. Each participant’s aligned diffusion data (FA, MD, RD, and AD) was then projected onto this skeleton. Demeaned *step error* and *variability* of unperturbed and perturbed steps were correlated against WM microstructural measures, both with and without age (demeaned) included as a covariate of no interest. For this, we used voxelwise cross-participant statistics (Randomize, 5000 permutations; Winkler et al. 2014) with threshold-free cluster enhancement (TFCE). TFCE is a method of finding clusters in the data (Smith and Nichols 2009). The Johns Hopkins University (JHU) tractography atlas was used to identify significant voxels.

## Results

### Behavioral results

#### Unperturbed precision stepping

**Fig. 2:**
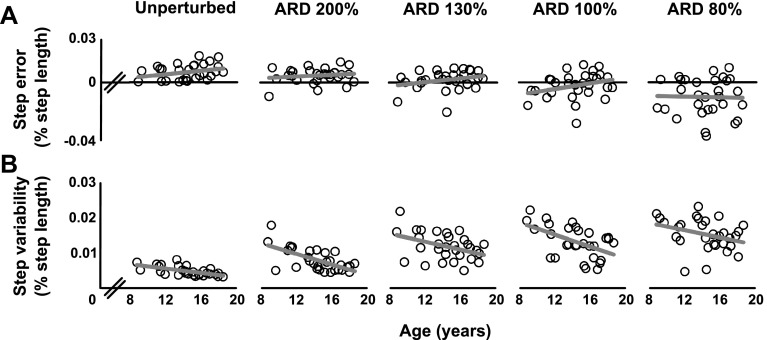
Precision stepping performance on unperturbed and perturbed step targets. Step error (**a**) did not significantly change with age or ARD (available response distance) in both unperturbed and perturbed precision stepping. Step variability (**b**) significantly decreased with age in both unperturbed and perturbed precision stepping. Moreover, a trend was found for increasing variability at shorter ARDs, albeit that these changes were non-significant. Each open circle represents a participant. Regression lines are displayed in grey

No correlations were found between *unperturbed step error* and age (*r*(29) = 0.300, *p* = 0.114), indicating that overall *unperturbed step error* was not different across the studied age span (Fig. 2a). However, step *variability* of unperturbed steps decreased with age (*r*(29) = − 0.618, *p* < 0.001). More specifically, older participants performed the task with higher consistency than younger participants (Fig. 2b).

#### Perturbed precision stepping

*Perturbed step error* was not different across participants of all ages (*F*_(1,28)_ = 0.729, *p* = 0.400) (Fig. 2a). The *step error* seemed to decrease at smaller ARD, but there was a large variability and the changes were not significant (*F*_(1.460, 40.885)_ = 1.557, *p* = 0.224). Moreover, no interaction between ARD and age was found (*F*_(1.460, 40.885)_ = 1.557, *p* = 0.224). For *variability* of perturbed trials, values decreased with age (*F*_(1,28)_ = 15.053, *p* = 0.001) (Fig. 2b). Moreover, no significant effect of ARD (*F*_(2.489, 69.687)_ = 0.904, *p* = 0.428) or interaction between ARD and age was found (*F*_(12.489, 69.687)_ = 0.607, *p* = 0.612) on step variability in perturbed trials. This indicated that the consistency of the foot placements onto the step targets in the perturbed condition was not significantly affected by decreasing ARDs (nevertheless, Fig. 2b clearly shows a tendency towards larger variability at smaller ARDs).

ARD did not significantly influence *perturbed step error* or *variability* (i.e., no main effect of ARD or age × ARD interaction was found). Guided by these behavioral results, the separate analyses of ARD conditions in combination with neural measures were no longer considered informative. Therefore, we subsequently averaged the step *error* and *variability* measures over all ARD conditions into two summary measures. These summary measures were subsequently fed into the statistical models used for detecting neural contributors to gait performance. By averaging across the ARD conditions, we were able to increase the statistical power and reduce the probability of Type I errors in these subsequent GM and WM analyses.

### Grey matter

**Fig. 3:**
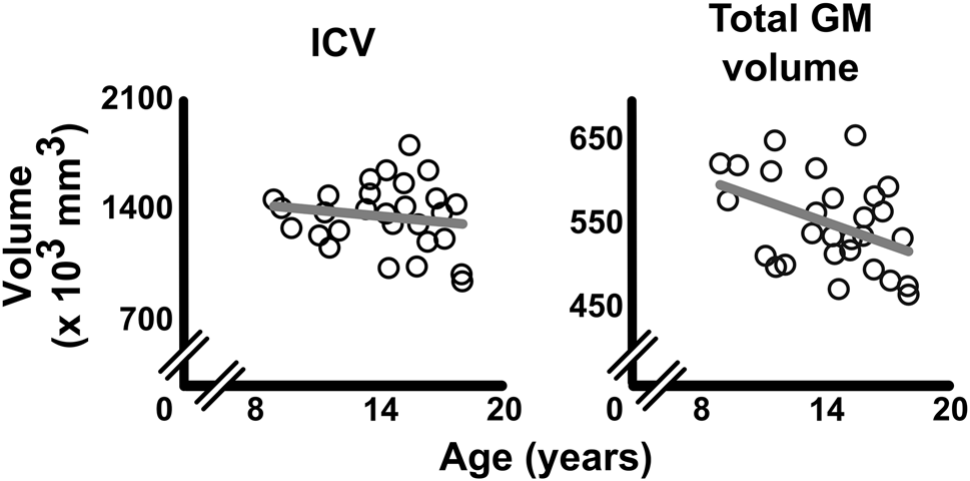
Age-related changes in ICV and total GM volume. Total intracranial volume (ICV) did not correlate with age (left), while whole-brain GM volume (right) did decrease with age (*r* = − 0.423, *p* = 0.025). Each open circle represents a participant. Correlation lines are displayed in grey

Total ICV did not correlate with age (*r*(26) = − 0.159, *p* = 0.420, Fig. 3), suggesting that maximal head size was reached in our sample (Lenroot and Giedd 2006). However, whole-brain GM volume decreased with age (*r*(26) = − 0.423, *p* = 0.025), confirming the generally observed ongoing maturation of GM during childhood (Lenroot and Giedd 2006) (Fig. 3).

No significant correlations were found between GM volumes of superparcels (frontal, parietal, temporal, occipital, and insula) from either the left or right hemisphere and *unperturbed* or *perturbed step error* or *variability* (*q* > 0.05), with or without controlling for ICV or age.

**Fig. 4:**
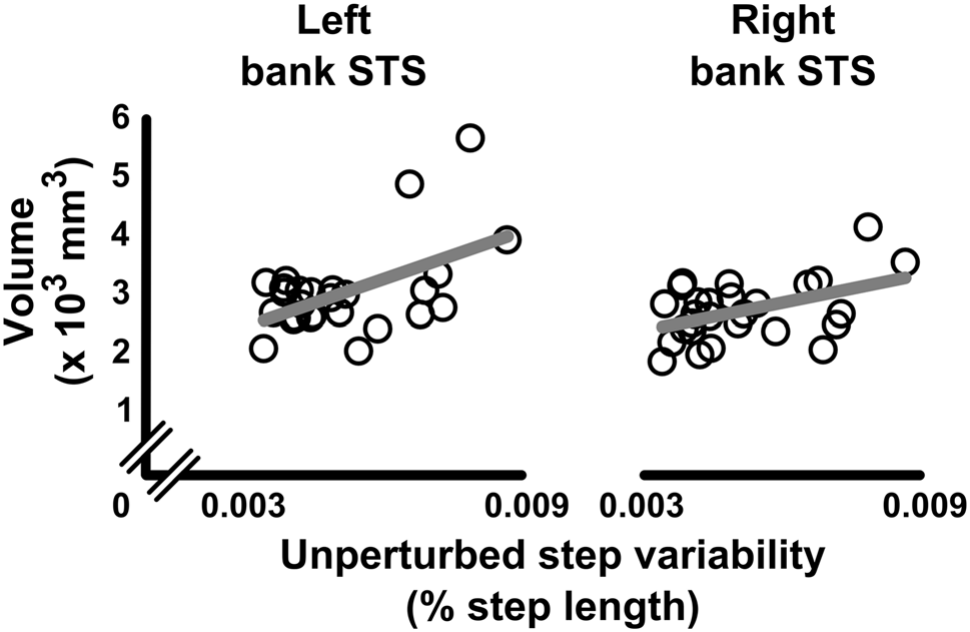
Relationships between unperturbed step variability and cortical GM volume of the banks of the STS. Lower cortical GM volumes of the banks of the superior temporal sulcus (bank STS) of both the left (*r* = 0.528, *p* = 0.005), and right (*r* = 0.461, *p* = 0.016) hemispheres were associated to lower unperturbed step variability. These correlations remained significant after controlling for ICV (left: *p* = 0.004; right: p = 0.002), but disappeared after controlling for age (*p* > 0.05). Each open circle represents a participant. Correlation lines are displayed in grey

When assessing the relationship between each parcel within a superparcel and step performance, *step variability* in *unperturbed precision stepping* showed significant positive correlations with GM volume of the banks of the superior temporal sulcus (bankSTS) in both the left (*r*(25) = 0.528, *p* = 0.005, *q* = 0.042) and right hemisphere (*r*(25) = 0.461, *p* = 0.016, *q* = 0.141) (Fig. 4). These correlations remained significant after controlling for ICV (left: *r*(24) = 0.551, *p* = 0.004, *q* = 0.032; right: *r*(24) = 0.589, *p* = 0.002, *q* = 0.014), but disappeared after controlling for age. *Step error* of *unperturbed precision stepping* showed a significant negative correlation with GM volume of the superior temporal cortex (*r*(25) = − 0.576, *p* = 0.002, *q* = 0.015) and insula (*r*(25) = − 0.383, *p* = 0.048, *q* = 0.048) in the right hemisphere. However, these associations disappeared after controlling for ICV or age. No significant associations were found between *step error of perturbed precision stepping* and parcel volumes (all *q* > 0.05).

### White matter

**Fig. 5:**
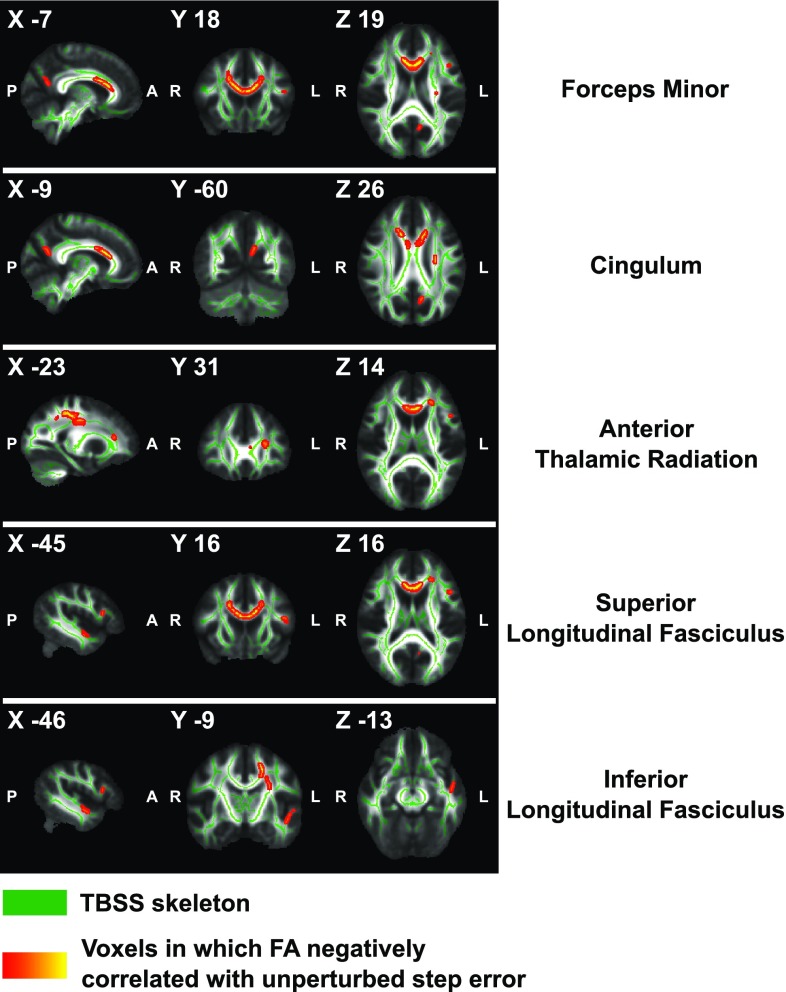
Negative correlations between fractional anisotropy and unperturbed step error. Voxels of which the FA values showed a significant negative correlation with unperturbed step error are displayed (red–yellow) onto a TBSS skeleton (green). These voxels resided in the forceps minor, left anterior thalamic radiation, left superior and inferior longitudinal fasciculus, and left cingulum. Labels by JHU White-matter Tractography Atlas. Results are controlled for age

**Fig. 6:**
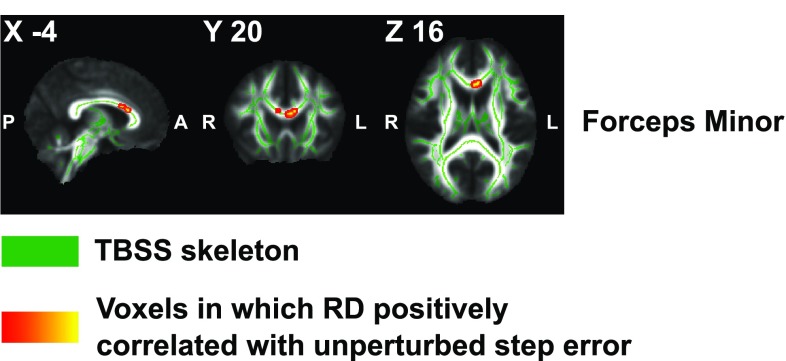
Positive correlations between radial diffusivity and unperturbed step error. Voxels of which the RD values showed a significant positive correlation with unperturbed step error are displayed (red–yellow) onto a TBSS skeleton (green). These voxels resided in the forceps minor. Labels by JHU White-matter Tractography Atlas. Results are controlled for age

Whole-brain voxelwise statistical analyses revealed associations between lower *unperturbed step error* and higher levels of FA in the forceps minor (FM; peak voxel MNI *x* = − 7, *y* = 18, *z* = 19), left anterior thalamic radiation (ATR; MNI − 23, 31, 14), left superior longitudinal fasciculus (SLF; MNI − 45, 16, 16), left inferior longitudinal fasciculus (ILF; MNI − 46, − 9, − 13), and left cingulum (MNI − 9, − 60, 26) (Fig. 5). When age was not controlled, associations between *unperturbed step error* and FA remained significant in voxels located within the forceps minor (MNI 5, 14, 20) and left ATR (MNI − 23, 31, 15). Furthermore, higher RD values in voxels within the forceps minor (MNI − 4, 20, 16) were related to larger *unperturbed step error* when age was controlled (Fig. 6). However, no significant correlations were found with RD values when age was not controlled.

MD and AD values showed no significant associations with *unperturbed step error* (with or without controlling for age). In addition, FA, MD, RD, and AD were not significantly associated with *perturbed step error*, or *(un)perturbed step variability* (with or without controlling for age).

## Discussion

This exploratory study aimed to investigate the potential relationship between structural brain properties and complex gait performance in youth aged 9–18 years. We utilized a treadmill-based precision stepping task to investigate complex gait performance, and volumetric analyses of cortical GM and whole-brain voxelwise statistical analyses of WM to investigate brain maturation. As hypothesized, (un)perturbed step variability decreased with age, yet there was no significant effect of the level of task complexity on perturbed step variability. Moreover, (un)perturbed step error was not significantly correlated with age or level of complexity. On the neural level, lower GM volumes of the banks of the superior temporal sulcus were associated with decreased unperturbed step variability, yet no GM associations were found for perturbed step accuracy. Furthermore, increased WM organization of tracts, connecting higher level brain regions, were associated with decreased unperturbed step error, beyond the general age effect. These results partly confirmed our hypothesis that advanced maturation of GM and WM structures is beneficial for precision stepping accuracy. However, our study was explorative in nature, and future studies are needed to confirm our findings.

### Neurobehavioral associations with step variability

As hypothesized, our results showed that the variability of foot placements on step targets decreases with age, both when the targets were placed on regular positions (unperturbed), and when they randomly shifted forwards (perturbed). These results support the findings of previous studies in healthy young participants (with ages ranging from 1 to 3 years to 10–17 years), showing decreases in variability of spatiotemporal gait parameters during basic gait (e.g., step length, step speed, double and single-support times) (Hausdorff et al. 1999; Vaughan et al. 2003; Dusing and Thorpe 2007; Froehle et al. 2013; Muller et al. 2013; Bisi and Stagni 2016; Gouelle et al. 2016; Manicolo et al. 2016). Moreover, during complex gait (e.g., obstacle avoidance tasks), age-related reductions have been reported for variability in foot clearances, take-off and landing distances, and muscular activations (McFadyen et al. 2001; Berard and Vallis 2006; Stern and Gottschall 2012; Corporaal et al. 2016). The origin of this motor variability may lie within any level of the motor pathway, from central processes (e.g., motor planning) to noise in peripheral processes (e.g., force production by muscles) (van Beers 2009; Dhawale et al. 2017).

In our study, we particularly focused on the possible role of the central processes (i.e., structural brain maturation), rather than on the peripheral components. Here, we hypothesized that structural GM and WM maturation of higher order brain regions would relate to improvements of step accuracy. Although results showed no relationship between WM organization and (un)perturbed step variability, regional GM volumes of the bilateral banks of the STS were positively associated with unperturbed step variability (but not with perturbed step variability; see “neural mechanisms of perturbed stepping”, below). Regarding the potential specific role of the STS in gait, functional MRI studies showed that the superior temporal sulcus deactivates during gait, as compared to quiet stance or gait imagery (Jahn et al. 2004; la Fougere et al. 2010). This deactivation is suggested to prevent unwanted movement corrections resulting from reflexive mechanisms for balance correction (Brandt et al. 1998; Jahn et al. 2004; la Fougere et al. 2010). As our precision stepping task was highly visually cued, it is plausible that brain regions regulating multisensory integration affected performance on this task. Moreover, the superior temporal sulcus is one of the latest regions in the temporal lobe to reach maturity (Lenroot and Giedd 2006). It is, however, important to note that the association between unperturbed step variability and GM volume of the banks of the STS disappeared when controlling for age. The banks of the STS lie within the multisensory vestibular cortex, together with the posterior insula, retroinsular region, and inferior parietal lobule (Dieterich and Brandt 2008), and are known to be involved in multisensory processing (i.e., vestibular, visual, auditory, and somatosensory information) (Calvert 2001; Wright et al. 2003; Beauchamp et al. 2004, 2008; van Atteveldt et al. 2004; Noesselt et al. 2007). Therefore, the effect of GM volume of the banks of the STS on gait variability might be better explained by general maturational processes, which are necessary to perform complex sensorimotor tasks, including complex gait. These GM maturational processes are thought to be driven by different biological processes, such as synaptic arborization, synaptic pruning, and axonal myelination (Lenroot and Giedd 2006). These processes generally increase the effectiveness of cortical neural processing. Although our volumetric analysis does not reveal which specific mechanism supports this GM change, it suggests a possible association between GM maturation of the STS and gait performance in our population.

It has been suggested that motor variability reflects the execution of a motor plan, rather than the creation of a motor plan, and would, therefore, originate from ‘noisy’ peripheral components, rather than central components of the motor system (Gouelle et al. 2013, 2016). This is in line with our results, showing no associations between step variability and structural brain differences in GM regions, or WM pathways, associated with motor planning (e.g., frontal regions, motor regions, and SMA). Possibly, peripheral sources contributed to age-related step variability in our sample such as noisy peripheral efferent and afferent pathways (Muller et al. 1994; McFadyen et al. 2001) or decreased corporeal awareness due to rapidly changing anthropometrics (Froehle et al. 2013). It is noteworthy, however, that structural brain maturation does not per se occur simultaneously with functional brain maturation (Supekar et al. 2010). For example, maturation of structural connectivity might be completed, while cortical activations and functional connectivity are still immature (Supekar et al. 2010; Cignetti et al. 2017). Therefore, inferences on functional associations between brain regions and precision stepping performance, based on our results should be made with caution.

### Neurobehavioral associations with step error

Although we hypothesized that step error would decrease with age, our results showed an absence of age-related differences in step error during both unperturbed and perturbed precision stepping. These findings seemingly contrast to the previous studies on other tasks involving complex gait, showing that gait strategies improved from childhood into adolescence (Pryde et al. 1997; McFadyen et al. 2001; Berard and Vallis 2006; Corporaal et al. 2016). Specifically, when children planned and executed their own avoidance strategies, age-related differences in gait patterns and avoidance success rates emerged (e.g., changes in step length, width, and speed, and foot placements and toe clearances relative to the obstacle). An important difference between these studies and our current experiment is that time pressure was present in the current study but much less in these previous studies. Moreover, these studies allowed free choice of avoidance strategies, and thus free choice of foot placements, while we constrained foot placements to predefined locations instead. Therefore, participants were not required to define a new, anticipatory gait strategy per se, but rather had to initiate fast goal-directed movements, based on external visual cues (i.e., the step targets).

Our results imply that the ability to perform goal-directed movements with the lower limbs is mature around the age of 8 years. Similarly, Choi et al. (2016) revealed stable, adult-like step accuracy on step targets in children aged 11–16 years (comparable to our sample of 9–18 years). These authors did, however, show decreasing step accuracy in younger children aged 6–10 years. This suggests that the maturation of processes underlying goal-directed movements of the lower limbs is not completed until approximately the age of 10 years. These results are consistent with the developmental trend found for manual goal-directed movements, showing that adult-like movements emerge around 8–11 years of age (Yan et al. 2000; Contreras-Vidal et al. 2005; Contreras-Vidal 2006; Favilla 2006; Kagerer and Clark 2014). Considering the findings of these studies, it may be suggested that cued goal-directed behavior (as used in precision stepping) matures earlier in life (around the age of 8–10 years) as compared to anticipatory planning behavior (e.g., free strategy selection during anticipated obstacle avoidance) which seems to mature at a more advanced age. Furthermore, we hypothesized that precision step error would decrease with more mature GM and WM brain structures. Results revealed no associations between GM volumes and step error in (un)perturbed precision stepping. WM microstructural organization, however, did reveal associations with unperturbed step error in the forceps minor, left anterior thalamic radiation, cingulum, and left superior, and inferior longitudinal fasciculus (but not for perturbed precision stepping error; see “Neural mechanisms of perturbed stepping”, below). Importantly, these results were particularly significant after controlling for age, which suggests that these brain–behavior associations were present beyond general maturation processes. These WM pathways are known to provide widespread reciprocal connections between several brain regions such as frontal, temporal, parietal, and occipital regions. Improved microstructural organization of these pathways has previously been associated with better performance on several spatiotemporal gait parameters (e.g., gait stability, stride length, single-support times, and gait speed), or specific gait tests (e.g., the timed up-and-go task or the number of steps while turning) in patients suffering from hydrocephalus and Alzheimer’s Disease (Marumoto et al. 2012) or Dementia (Scherder et al. 2011) and in older adults (Scherder et al. 2011; Marumoto et al. 2012; Bolandzadeh et al. 2014; Bruijn et al. 2014; Verlinden et al. 2016; Seiler and Pirpamer 2017). For example, the forceps minor (connecting bilateral prefrontal cortices) has previously been associated with gait speed (Seiler and Pirpamer 2017) and abnormal gait scores on a Tinetti gait assessment (Bhadelia et al. 2009) in older adults. In addition, these WM pathways have been associated with several cognitive functions. The forceps minor has been associated with visuomotor speed, memory, and executive function (Biesbroek et al. 2016), the SLF with attention, visuospatial ability, and sensorimotor integration (Makris et al. 2005; Turken et al. 2008; Scherder et al. 2011; Vestergaard et al. 2011; Chaddock-Heyman et al. 2013; Klarborg et al. 2013; Kamali et al. 2014; Rodriguez-Herreros et al. 2015; Urger et al. 2015; Amemiya and Naito 2016), and the ATR with spatial information processing, movement initiation, and planning (Scherder et al. 2011).

Since end-point accuracy of precision steps has been shown to require profound visuospatial attention (e.g., Smid and den Otter 2013; Hollands et al. 1995), it is, therefore, plausible that tracts involved in such functions show associations with precision step error in our task (Patla and Vickers 1997, 2003; Higuchi 2013; Koenraadt et al. 2014). Importantly, the WM associations with unperturbed precision step error that were found in our study were particularly present after we controlled for age-related variations in WM characteristics (i.e., by including age as covariate of no interest). This demonstrates that these associations were possibly not driven by WM differences related to age, but rather to normal WM heterogeneity within the population. These results, therefore, substantiate the assumption that age-related structural brain differences do not primarily predict goal-directed precision stepping performance in our sample. It has been shown, however, that age-related structural brain changes are ongoing in the age range considered in this study (Giedd et al. 1999; Gogtay et al. 2004; Lenroot and Giedd 2006; Raznahan et al. 2011; Yap et al. 2013; Ducharme et al. 2016) and it can, therefore, not be ruled out completely that structural brain maturation has no (direct or mediating) role in gait performance. For instance, in children aged 5–17 years, age-related changes in the SLF have been associated with improved cognitive functions such as set-shifting and attention (Urger et al. 2015), which is assumed to play a role in the visually cued task examined here. Functional compensatory mechanisms may attenuate the relationship between structural maturity and behavioral outcome, such as in older adults (Heuninckx et al. 2008; Goble et al. 2010). Future studies may shed light on these possible compensatory mechanisms in children.

### Neural mechanisms of perturbed stepping

We hypothesized that the impact of age-related structural brain changes in higher order cortical regions on stepping accuracy would be stronger in perturbed as compared to unperturbed precision stepping. As briefly mentioned above, however, we found no associations between GM or WM maturation and perturbed precision stepping accuracy, whereas we did for unperturbed stepping.

A recent review on the neural control of fast gait adjustments by Potocanac and Duysens (2017) suggested that fast motor responses to perturbations in the environment likely engage different neural mechanisms than unperturbed environments (Weerdesteyn et al. 2004; Reynolds and Day 2005; Potocanac et al. 2014; Hoogkamer et al. 2015; Mazaheri et al. 2015). For instance, Weerdesteyn et al. (2004) showed that when sudden obstacles had to be avoided, this provoked faster than voluntary responses, suggesting involvement of ‘fast’ subcortical rather than ‘slow’ cortical pathways. This was substantiated by findings of Marigold et al. (2007), who showed that re-direction of visual gaze towards an obstacle does not always occur when responding to its unpredictable appearance. These findings suggest that the production of fast stepping responses may require less cortical processing (e.g., of visuospatial information) than preplanned stepping responses. Possibly, other neural mechanisms (e.g., subcortical) may be involved. If these observations translate to our precision stepping task, it may be that the impact of maturational processes of cortical brain structures is smaller for perturbed as compared to unperturbed precision stepping.

Although the present data may provide evidence for a role of subcortical processing, they should, however, not be taken as evidence that online step adjustments are entirely independent of cortical processing. It should be pointed out that online corrections normally involve a mixture of subcortical and cortical pathways. Cortical areas, such as the posterior parietal cortex, may play a supervisory role over faster subcortical routes (Glickstein 2003; Reynolds and Day 2012). In addition, there is evidence for fast cortical processing (not reaching conscious levels; see Potocanac and Duysens 2017). Possibly, these cortical involvements in online step adjustments are not of primary importance in the age group investigated in the present study. In young adults (around 25 years of age), the addition of a dual task barely affected performance in a similar perturbed precision stepping task (Mazaheri et al. 2015). In contrast, this task was more difficult to perform in older as opposed to younger adults when the task was combined with a dual task (relying on cortical resources; Mazaheri et al. 2014, 2015). It thus appears that there is a shift towards more cortical involvement in aged groups. If this also applies to young participants, increased cognitive involvement may overcome the adverse effects of suboptimal cortical structures on behavior, similar to compensatory mechanisms on motor control in older adults (Heuninckx et al. 2008; Goble et al. 2010). Future studies may be able to shed light on the interplay between functional and structural cognitive involvement in the online adjustments of steps.

## Limitations

This study aimed to explore the relationship between structural brain maturation and complex gait performance in a population of 9–18 years. It has to be noted that many developmental changes take place during this age period. Aside from structural brain maturation, for example, also functional brain development is ongoing. Furthermore, lower levels of the CNS are developing and the musculoskeletal system is changing. These changes may decrease corporeal awareness due to rapidly changing anthropometrics (Froehle et al. 2013). Although we controlled for differences in body size by normalizing our gait parameters to individual’s body proportions (Hof 1996; Vaughan et al. 2003), we cannot completely rule out the possibility that developmental processes other than structural brain properties may have influenced our results. On the other hand, structural brain maturation may have impacted other sensorimotor processes, which (in)directly affected gait performance. Thus, complex sensorimotor coordination, irrespective of the specific motor task, should be investigated beyond the typical structural brain maturation analyses. Therefore, our results should be interpreted with caution, and should be used as preliminary evidence for a potential relationship between gait maturation and brain maturation. Future research may adopt an approach in which multiple developmental processes are considered as potential mediators for gait development.

## Conclusion

Both unperturbed and perturbed precision stepping mature surprisingly fast as expressed by an absence of significant age-related changes in step error. Variability, however, does decrease with age and this may indicate the presence of slower maturational processes. In addition, the present study has provided, for the first time, preliminary insights into the potential link between structural brain properties and precision stepping performance in youth. For unperturbed precision stepping, the precision step error was associated with WM microstructural organization of pathways involved in attentional and visual processing. Such correlations were absent for perturbed precision stepping, requiring online adjustments of steps. This difference is consistent with the viewpoint that the neural control may differ, in particular pointing to a dominant role for fast pathways in online corrections, possibly involving subcortical circuits.

## Abbreviations

AD: Axial diffusivity
ARD: Available response distance
ATR: Anterior thalamic radiation
COP: Center of pressure
FA: Fractional anisotropy
FDR: False discovery rate
GM: Grey matter
ICV: Intracranial volume
MD: Mean diffusivity
MNI: Montreal neurological institute common coordinate space
RD: Radial diffusivity
ROI: Regions of interest
SLF: Superior longitudinal fasciculus
STS: Superior temporal sulcus
WM: White matter
